# Perioperative Antibiotics in Appendicitis—Do We Need to Adjust Therapy for the Elderly? A Matched Pair Analysis

**DOI:** 10.3390/antibiotics11111525

**Published:** 2022-11-01

**Authors:** Jens Strohäker, Martin Brüschke, Nora Leser, Alfred Königsrainer, Ruth Ladurner, Robert Bachmann

**Affiliations:** Department of General, Visceral and Transplantation Surgery, University Hospital of Tuebingen, 72076 Tuebingen, Germany

**Keywords:** complicated appendicitis, elderly, perioperative antibiotics, antibiotic stewardship

## Abstract

(1) Background: Perioperative Antibiotics for acute complicated appendicitis are a standard of care. While there are plenty of trials for pediatric patients, data for elderly patients are scarce. The goal of our study was to evaluate whether elderly patients carry more resistant bacteria and thus have less favorable outcomes after an appendectomy that may warrant intensified perioperative antibiotic treatment (2) We present a retrospective single-center matched pair (139 patients each) analysis of perioperative and microbiological outcomes of an elderly appendicitis cohort (i.e., older than 60 years) compared with a younger adult cohort (i.e., ≤60 years). Both groups were matched one for one according to gender, duration of symptoms, c-reactive protein at presentation and whether they presented with uncomplicated or complicated appendicitis. (3) Results: After matching, complicated appendicitis was present in 76.3% of both groups. Elderly patients more frequently received preoperative diagnostic CT (*p* < 0.001) than the young. Both operative strategy (laparoscopic appendectomy in 92.1% each) and duration of surgery (57 vs. 56 min) were equal in both groups. Postoperative antibiotics were prescribed in ~57% for a median of 3 days in both groups and antibiotic selection was similar. The incidence of surgical site infections was higher in the young (12.2% vs. 7.9%) yet not significant. There was no difference in culture positivity or bacterial spectrum and the elderly cohort did not present with increased resistant bacterial isolates. (4) Conclusions: While overall resistant bacterial strains were rare, perioperative outcomes between the young and the elderly did not differ and did neither warrant longer nor intensified antibiotic treatment.

## 1. Introduction

Acute appendicitis is among the most commonly treated diseases in general surgery. Preoperative antibiotics are recommended to lower the incidence of postoperative surgical site infections [1]. Usually, source control by appendectomy is sufficient for the termination of antibiotic therapy. While additional antibiotics are generally not recommended [2,3], they are frequently prescribed and may have additional merit [4,5]. Preoperative antibiotics appear to successfully slow down disease progression to safely delay surgery [6]. In recent decades, data became available regarding the safety and efficacy of purely conservative (i.e., antibiotic) treatment for uncomplicated appendicitis [7]. Non-operative treatment success was usually achieved with intravenous broad-spectrum antibiotics [8,9]. With the increase in the world population, the elderly patient cohort is expected to grow steadily over the next 30 years. By 2050, the number of people aged 60 years and older will match the cohort of those under the age of 15 at two billion people each [10]. While there are plenty of randomized controlled trials regarding the treatment of appendicitis in the pediatric population, prospective and/or randomized trials specifically targeting the elderly are missing. Most treatment recommendations are based on retrospective data. It was not until 2020 that the first guidelines targeting appendicitis in the elderly were published [11].

The threshold for the use of broad-spectrum antibiotics appears lower in the elderly, who are frailer and at risk for complications. Additionally, they have been shown to carry more resistant bacterial strains [12]. There are no data from randomized trials, however, that support this practice in the context of appendicitis. The goal of this study was to evaluate whether there are differences in the bacterial spectrum and the isolates’ resistance patterns as well as differences in treatment algorithms and patients’ outcomes between a young (i.e., ≤60 years of age) and an elderly (>60 years of age) cohort at a tertiary central European teaching hospital.

## 2. Results

### 2.1. Clinical Characteristics

Between 2014 and 2021, 1193 patients underwent surgery for acute appendicitis at our tertiary teaching hospital. We performed one to one case–control matching (for matching criteria see Methods, Section 4.2) and ultimately included two cohorts of patients with 139 patients each in our analysis. The “young” cohort was made up of adult patients (i.e., 16 and older) with a maximum age of 60. The mean age was 38.4 years compared with 69.8 years in the elderly group. Given the difference in age, there was a significant difference in the American Association of Anesthesiologists (ASA) Scores of both groups (*p* < 0.001). The diagnostic approach with respect to imaging was also significantly different. In the young cohort, nine out of ten patients underwent diagnostic ultrasound and about a third of patients had an (additional) computed tomography (CT). The elderly group had significantly fewer ultrasounds (73.4% *p* < 0.001) and twice as often a CT than the young group (69.1% *p* < 0.001). The surgical approach (laparoscopic vs. open appendectomy) was similar between both groups (*p* = 0.487) as was the median operating time of 57 vs. 56 min (*p* = 0.710). The median length of stay was longer in the elderly group (3 vs. 2 days) although the difference was not statistically significant. The rate of surgical site infections was higher in the young cohort at 12.2%, compared with 7.9%. This difference was also not statistically significant. For details, see Table 1.

### 2.2. Perioperative Patient Management

According to the matching process, 106 patients (76.3%) in each cohort were classified as complicated appendicitis. Intraoperative cultures were drawn at the discretion of the lead surgeon and there was no standardized protocol regarding the indication for microbiological testing. Overall, 94 of the young patients had intraoperative cultures drawn compared with 95 intraoperative cultures in the elderly cohort. Culture positivity was comparable in both groups (77.7 vs. 81.1%, *p* = 0.564). Postoperative antibiotic continuation, which exceeded the preoperative single-shot antibiotic, was similar between both groups (56.8 vs. 57.6%, *p* = 0.904). While the median duration of antibiotic treatment post-operatively was 3 days in both cohorts the interquartile range (IQR) was higher in the elderly cohort, although the difference was not significant (*p* = 0.095). The empiric antimicrobial agent selection was similar with a trend towards intravenous and broad-spectrum substances (piperacillin/tazobactam in particular) in the elderly cohort. For details, see Table 2.

### 2.3. Microbiological Results

**Figure 1:**
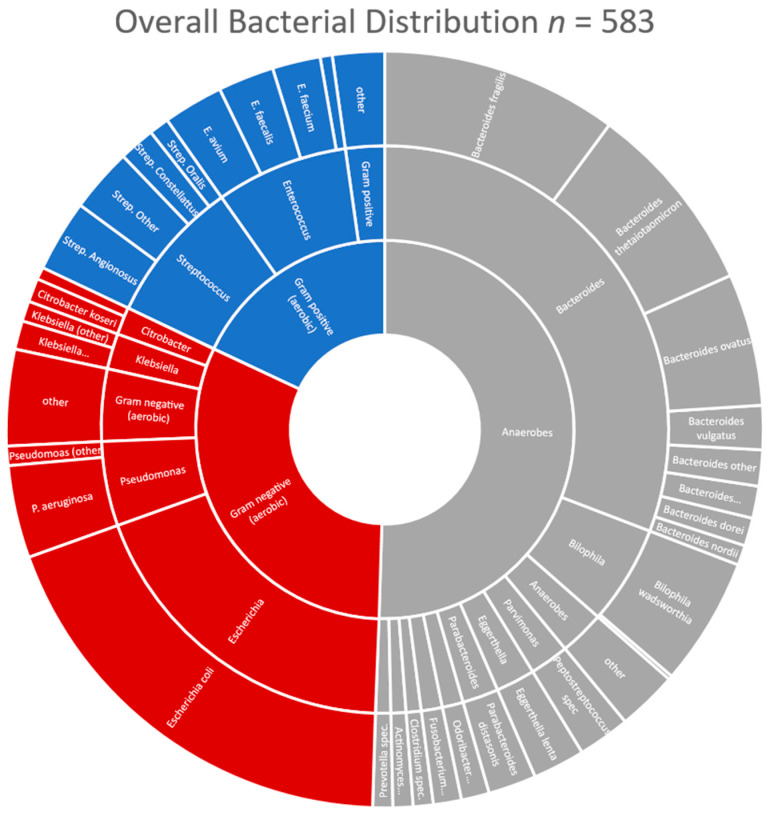
Overall Bacterial Distribution (strict anaerobes grey, Gram-negative bacteria red, Gram-positive blue).

Of the 189 intraoperative cultures, 150 yielded bacterial growth (79.4%). Most of the positive cultures were polymicrobial with up to ten different bacterial strains. Overall, 583 bacteria were cultured and equally distributed between the two groups (291 in the young cohort vs. 292 in the elderly cohort). Strict anaerobic bacteria made up 50.6% of all isolates. Bacteroides represented the largest bacterial genus with 180 isolates. Escherichia coli was the most common individual bacterial strain (*n* = 111). Forty-four isolates of Enterococcus species were isolated with equal distribution between cohorts (22 each). Enterococcus avium was the most common, closely followed by *E. faecalis* and *E. faecium*. There was a single stain of vancomycin-resistant *E. faecium* in the entire study population. Pseudomonas was isolated in 28 cultures without a single resistant strain. Stenotrophomonas was represented in four cultures and Acinetobacter baumannii in one culture. These healthcare-associated pathogens needed not be addressed specifically. While we did not perform anaerobic antibiotic resistance testing for each anaerobe, we recently experienced increasing piperacillin/tazobactam resistance in Bacteroides thetaiotaomicron and Bacteroides ovatus isolates with up to 20% resistance to the acylureido-penicillin. However, these at-risk Bacteroid strains were also equally distributed between the two cohorts (45.8% vs. 44.0%, *p* = 0.810). An overview of the bacterial distribution can be found in Figure 1.

### 2.4. Antimicrobial Resistances

All (facultative) aerobic bacteria isolated from intraoperative material were cultured by the local microbiology department and tested for antibiotic resistance according to the European Society of Clinical Microbiology and Infectious Diseases (EUCAST) guidelines. Strictly anaerobic bacteria, per protocol, only undergo antibiotic resistance testing when found in monocultures, which was rarely the case (*n* = 1). Resistance breakpoints were defined according to the EUCAST guidelines. Susceptibility to a high-dose regimen was classified as susceptible. Bacteria that are always resistant to specific substances were defined as intrinsically resistant. Bacteria that may be susceptible to an antibiotic substance but were tested resistant to that substance were classified to have acquired said resistance. Acquired resistance was classified as resistant whereas intrinsic resistance was marked separately.

#### 2.4.1. Aerobic Gram-Positive Bacteria

**Figure 2:**
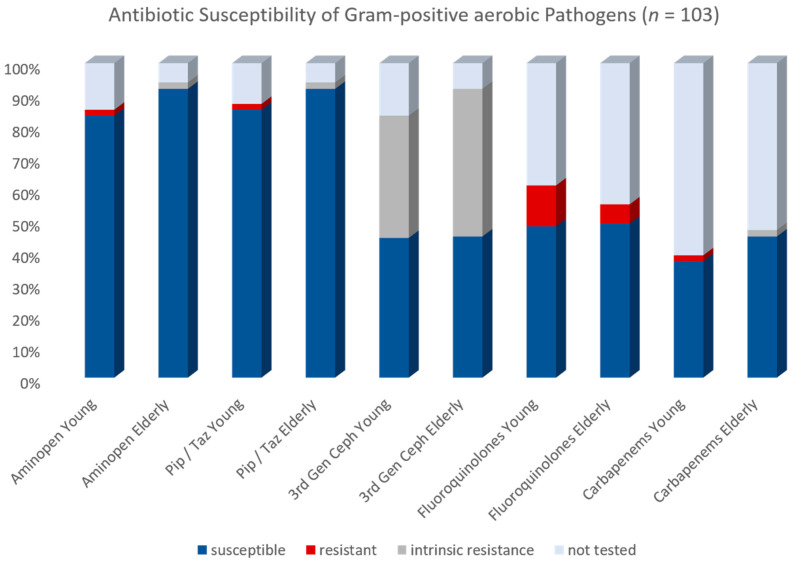
Antibiotic Susceptibility of Gram-positive aerobic Pathogens.

Microbiological testing yielded 103 Gram-positive aerobic isolates. Overall antibiotic resistance rates were low. Standard resistance testing consisted of the six following antibiotic classes: aminopenicillin (usually ampicillin/sulbactam), acylureido-penicillin (usually piperacillin/tazobactam), cephalosporins of the 3rd generation (usually cefotaxime and/or ceftriaxone) carbapenems (usually meropenem), fluoroquinolones (usually ciprofloxacin) and glycopeptides (usually vancomycin). There were some variations for specific bacteria (e.g., no cephalosporin testing for enterococcus spec.). For the Gram-positive bacteria, aminopenicillin susceptibility was 87.3% with an acquired resistance rate of 0.9%, an intrinsic resistance of 0.9% and omission of testing in 10.6%. The acylureido-penicillin susceptibility was 88.3% with an acquired resistance rate of 0.9% an intrinsic resistance of 0.9% and an omission of testing in 9.7%. The third-generation cephalosporin susceptibility was 44.7% with an acquired resistance rate of 0.0%, an intrinsic resistance of 42.7% (all enterococcus) and an omission of testing in 12.6%. Carbapenem susceptibility was 40.8% with an acquired resistance rate of 0.9%, an intrinsic resistance of 0.9% and an omission of testing in 57.3%. Fluoroquinolone susceptibility was 48.5%, with an acquired resistance rate of 9.7% and no intrinsic resistance. In 41.7%, no resistance testing for fluoroquinolones was performed (mainly streptococcus). Glycopeptide susceptibility was 96.1% with 2.9% acquired and 0.9% intrinsic resistance. We compared the antimicrobial resistance rates between the two cohorts. There was no difference for the aminopenicillin (*p* = 0.207), acylureido-penicillin (*p* = 0.325), cephalosporins (*p* = 0.394), carbapenems (*p* = 0.423) and fluoroquinolones (*p* = 0.481). There was also no difference for the glycopeptide vancomycin (*p* = 0.244). For a graphical distribution see Figure 2.

#### 2.4.2. Aerobic Gram-Negative Bacteria

**Figure 3:**
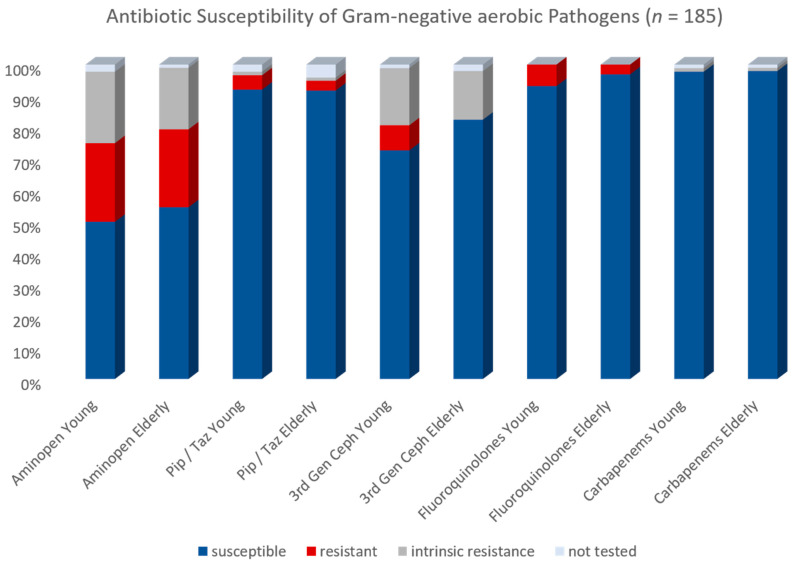
Antibiotic Susceptibility of Gram-negative aerobic Pathogens.

Microbiological testing yielded 185 Gram-negative aerobic isolates. Overall antibiotic resistance rates were low. Standard resistance testing consisted of the five following antibiotic classes: aminopenicillin (usually ampicillin/sulbactam), acylureido-penicillin (usually piperacillin/tazobactam), cephalosporins of the 3rd generation (usually cefotaxime and/or ceftriaxone) carbapenems (usually meropenem) and fluoroquinolones (usually ciprofloxacin). There were some variations for specific bacteria (e.g., ceftazidime testing for pseudomonas). For the Gram-negative bacteria, aminopenicillin susceptibility was 52.4% with an acquired resistance rate of 24.9%, an intrinsic resistance of 21.1% and an omission of testing in 1.6%. The acylureido-penicillin susceptibility was 91.9% with an acquired resistance rate of 3.8% an intrinsic resistance of 1.1% and an omission of testing in 3.2%. The 3rd generation cephalosporin susceptibility was 77.8% with an acquired resistance rate of 3.8%, an intrinsic resistance of 16.8% (all pseudomonas) and omission of testing in 1.6%. Carbapenem susceptibility was 97.8% with an acquired resistance rate of 0.0%, an intrinsic resistance of 1.1% and an omission of testing in 1.1%. Fluoroquinolone susceptibility was 95.1%, with an acquired resistance rate of 4.9% and no intrinsic resistance. We compared the antimicrobial resistance rates between the two cohorts. There was no difference for the aminopenicillin (*p* = 0.836), acylureido-penicillin (*p* = 0.875), carbapenems (*p* = 1.000) and fluoroquinolones (*p* = 0.313). For the cephalosporins, we observed all seven cases of acquired resistance in the young cohort with an otherwise equal distribution of intrinsic resistance and a correspondingly higher percentage of susceptible strains in the elderly. This difference was statistically significant (*p* = 0.017). For a graphical distribution see Figure 3.

## 3. Discussion

The elderly patient population is growing and in 2050 people over 60 years are expected to make up 21% of the world’s population [10]. While appendicitis is certainly a disease most frequently encountered in younger patients, this will also lead to an increased incidence in elderly patients. For the pediatric population there are plenty of trials focusing on the management of appendicitis in our youngest but near to no trials focusing on the opposite of the age spectrum. Only recently the first guidelines were published targeting the management of appendicitis in patients over 65 and it is mainly referring to data not specifically collected for this population [11].

To the best of our knowledge, we present the first analysis of specific microbiological results and infectious disease outcomes of elderly patients with appendicitis. We, therefore, performed a retrospective one-for-one matched pair analysis for a group of 139 patients older than 60 years matched to a group of 139 individuals of up to 60 years of age undergoing appendectomy for appendicitis.

We hypothesized that the elderly cohort may have been diagnosed and treated differently and may have suffered from more complex infections due to acquired bacterial resistance secondary to prior antibiotic therapies and or consumption of meat from livestock fed with antibiotics [13]. Our two cohorts were matched exactly for gender, duration of symptoms, c-reactive protein at presentation and classification of uncomplicated/complicated disease. While most cases of appendicitis generally represent uncomplicated disease, we observed that in the elderly, complicated cases were far more common. Complicated appendicitis has been reported to be present in up to 70% of seniors [11]. In our prospective database even more than 75% of patients over the age of 60 suffered from complicated appendicitis. This was also represented in the two study cohorts after the matching process. We observed trends to treat elderly patients longer and with broader antibiotics although these were not significant. The outcomes of both cohorts were rather favorable. Surgical Site Infections developed in 12.2% of young and 7.9% of elderly patients. While this number is higher than usual, one needs to keep in mind the 76% of complicated cases in our study population. Younger age has been described as a potential risk factor for SSI which is in accordance with our findings [14].

The bacterial spectrum encountered in both cohorts was near identical with similar numbers of cultures drawn, culture positivity and bacteria. Interestingly age itself was no predictor of more resistant bacterial strains in our study. There were near to no differences in the microbial resistance patterns between the two groups. There is controversy among different reports that recently reported increased bacterial resistance to be directly dependent on patient age [15,16,17]. This was studied mainly in E. coli and Staph aureus. Additionally, most frequently the phenomenon was reported for fluoroquinolones.

The empiric use of broad-spectrum antibiotics in acute uncomplicated appendicitis has been studied in a post hoc analysis of the EAST-Mustang Trial. The authors reported no difference in SSI and intraabdominal abscesses between narrow and broad-spectrum antibiotics [4]. For complicated appendicitis, there are no comparable trials. A 2018 study by Obayashi et al. reported no benefit from broad-spectrum antibiotics in pediatric complicated appendicitis; however, their antibiotic selection was rather unstandardized and they did not report the frequency of complicated appendicitis but rather reported low-risk and high-risk groups [18].

Until proper trials exist, clinicians are forced to rely on data collected from retrospective analyses or from trials not focusing on that elderly cohort. Elderly patients are at increased risk of complications due to antimicrobial side effects or drug interactions [19]. The acylureido-penicillin piperacillin/tazobactam is a generally well tolerated broad-spectrum and pseudomonas-covering antimicrobial and is frequently prescribed in perforated appendicitis for its coverage of both pseudomonas and enterococcus. However, it is accompanied by the risk of acute kidney injury in elderly patients both when used as a single agent (up to 7%) or in combination with vancomycin (up to 12%) [20]. In the light of our results, we cannot support the use of broad-spectrum antibiotics in not severely ill patients with complicated appendicitis neither for young nor elderly patients. In our study carbapenems would have had the best overall single-agent coverage, followed by piperacillin/tazobactam. However, given the low resistance rates to ceftriaxone and metronidazole and the overall low rate of pseudomonas and enterococcus encountered, a combination of cephalosporin plus metronidazole should be suitable for most patients. Fluoroquinolones, while having an excellent coverage of Gram-negative bacteria should generally be avoided due to the risk of side effects, especially in elderly patients [21]. The generalization of our results is of course limited by the overall low rate of ESBL/MRGN and VRE in our region.

## 4. Materials and Methods

### 4.1. Data Acquisition

We retrospectively screened our hospital information system for all patients who underwent an appendectomy or ileocecal resection for acute appendicitis at our tertiary Department of General, Visceral and Transplantation Surgery. All consecutive adult patients (age ≥ 16 years) who underwent an open or laparoscopic appendectomy or ileocecal resection for appendicitis between January 2014 and February 2021 were entered into a database. Patients that had an appendectomy as part of another procedure were excluded as well as all patients that underwent non-acute appendectomy (chronic appendicitis and tumors). In total 1193 patients entered into the matching process.

### 4.2. Matching Process

In the overall cohort, around 33% of young patients were classified as complicated appendicitis compared with around 75% of elderly patients. In order to properly compare the two cohorts, we performed 1:1 case–control matching. The five criteria chosen for the matching process were: Gender, Duration of Symptoms in Days, Classification as complicated or uncomplicated appendicitis, and c-reactive protein (CRP) on initial presentation. For this process, CRP was grouped into 5 different groups (CRP < 2.5 mg/dL; CRP between 2.5 and 5 mg/dL; CRP between 5 and 7.5mg/dL; CRP between 7.5 and 10 mg/dL and CRP > 10 mg/dL). All four criteria had to match exactly, without tolerance.

### 4.3. Clinical Definitions

Uncomplicated and complicated appendicitis was classified according to the European Association of Endoscopic Surgery (EAES) Consensus Guidelines [2]. Complicated Appendicitis was defined as the presence of gangrenous appendicitis, perforation of the appendix, local abscess or peritonitis.

### 4.4. Isolation and Identification of Strains

All microbiological samples were sent for both aerobic and anaerobic cultures. For the anaerobic cultures, a custom-built highly enriched and supplemented sheep-blood agar was used. The exact method has been described by the local microbiology department [22]. The isolates were identified using matrix-assisted laser desorption time-of-flight–mass spectrometry.

### 4.5. Statistics

Comparison between groups was carried out by Chi-squared test or Fisher’s exact test for nominal variables, and Mann–Whitney U test or Kruskal–Wallis test for non-normal distributed continuous variables, as appropriate. A probability of less than 0.05 was considered to be statistically significant. All *p*-values reported are results of two-sided testing. Where needed, Bonferroni correction was applied. Statistical analysis was carried out using IBM SPSS Statistics for Windows, Version 28.0 (IBM Corp., Armonk, NY, USA).

## 5. Conclusions

Appendicitis in the elderly will become an increasing topic over the next thirty years. Until proper trials are available, the perioperative management will be based on current guidelines and retrospective analyses. In the absence of other risk factors or known carrier status of resistant bacteria, complicated appendicitis in the elderly does not warrant a prolonged or broadened spectrum of perioperative antibiotics.

## Abbreviations

ASAAmerican Society of AnesthesiologistCRPC-reactive proteinCTComputed tomographyEAESEuropean Association of Endoscopic SurgeryEUCASTEuropean Society of Clinical Microbiology and Infectious DiseasesIQRInterquartile rangeSDStandard deviationSSISurgical site infection 

## Figures and Tables

**Table 1:** Baseline characteristics.

	Young (*n* = 139)	Elderly (*n* = 139)	*p*
Sex male (Percentage)	68 (49%)	68 (49%)	1
Age (mean in years ± SD)	38.4 (±13.6)	69.8 (±7.5)	0
Body mass index (mean in kg/m^2^ ± SD)	25.9 (±5.6)	27.1 (±5.2)	0.019
American Society of Anesthesia-Score			
I	77 (55.4%)	13 (9.4%)	<0.001
II	53 (38.1%)	95 (68.3%)	
III	9 (6.5%)	31 (22.3%)	
Preoperative diagnosis			
Ultrasound	126 (90.6%)	102 (73.4%)	<0.001
Computed tomography	49 (35.3%)	96 (69.1%)	<0.001
Complicated Appendicitis	106 (76.3%)	106 (76.3%)	1
C-reactive protein at presentation			1
<2.5 mg/dL	31 (22.3%)	31 (22.3%)	
2.5–5 mg/dL	19 (13.7%)	19 (13.7%)	
5–7.5 mg/dL	11 (7.9%)	11 (7.9%)	
7.5–10 mg/dL	10 (7.2%)	10 (7.2%)	
>10 mg/dL	68 (48.9%)	68 (48.9%)	
Operating time (median in min, range)	57 (16–211)	56 (18–186)	0.71
Surgical strategy			0.487
Open appendectomy	6 (4.3%)	3 (2.2%)	
Laparoscopic appendectomy	128 (92.1%)	128 (92.1%)	
Conversion to open appendectomy	5 (3.6%)	8 (5.8%)	
Length of stay (median in days, range)	2 (1–25)	3 (1–15)	0.159

**Table 2:** Infectious disease outcomes.

	Young (*n* = 139)	Elderly (*n* = 139)	*p*
Intraoperative cultures drawn	94 (67.6%)	95 (68.3%)	0.898
Intraoperative culture positivity	73 (77.7%)	77 (81.1%)	0.564
Postoperative antibiotic continuation	79 (56.8%)	80 (57.6%)	0.904
Duration of postoperative antibiotics (median in days, IQR)	3 (2–4)	3 (3–5)	0.095
Antibiotic classes/substance			
Aminopenicillins	2 (0.1%)	1 (0.1%)	
Piperacillin/tazobactam	23 (16.5%)	35 (25.2%)	
2nd Generation cephalosporins	2 (0.2%)	3 (2.2%)	0.387
3rd Generation cephalosporins	25 (18.0%)	19 (13.7%)	
Fluoroquinolones	26 (18.7%)	22 (15.8%)	
Carbapenems	0	0	
± Metronidazole	52 (37.4%)	45 (32.4%)	0.216
Surgical site infection	17 (12.2%)	11 (7.9%)	0.232

## Data Availability

The data presented in this study are available on request from the corresponding author. The data are not publicly available due to the pseudonymized character of the data.
